# Anti-MUC1 Monoclonal Antibody (C595) and Docetaxel Markedly Reduce Tumor Burden and Ascites, and Prolong Survival in an *in vivo* Ovarian Cancer Model

**DOI:** 10.1371/journal.pone.0024405

**Published:** 2011-09-09

**Authors:** Li Wang, Hongmin Chen, Mohammad H. Pourgholami, Julia Beretov, Jingli Hao, Hongtu Chao, Alan C. Perkins, John H. Kearsley, Yong Li

**Affiliations:** 1 Department of Gynecologic Oncology, Henan Cancer Hospital, Zhengzhou, Henan, China; 2 Faculty of Medicine, University of New South Wales, Sydney, Australia; 3 Department of Surgery, St George Hospital, Sydney, Australia; 4 Cancer Care Centre, St George Hospital, Sydney, Australia; 5 Anatomical Pathology, St George Hospital, Sydney, Australia; 6 Academic Medical Physics, Medical School, Queen's Medical Centre, Nottingham, United Kingdom; University of Chicago, United States of America

## Abstract

MUC1 is associated with cellular transformation and tumorigenicity and is considered as an important tumor-associated antigen (TAA) for cancer therapy. We previously reported that anti-MUC1 monoclonal antibody C595 (MAb C595) plus docetaxel (DTX) increased efficacy of DTX alone and caused cultured human epithelial ovarian cancer (EOC) cells to undergo apoptosis. To further study the mechanisms of this combination-mediated apoptosis, we investigated the effectiveness of this combination therapy *in vivo* in an intraperitoneal (i.p.) EOC mouse model. OVCAR-3 cells were implanted intraperitoneally in female athymic nude mice and allowed to grow tumor and ascites. Mice were then treated with single MAb C595, DTX, combination test (MAb C595 and DTX), combination control (negative MAb IgG_3_ and DTX) or vehicle control i.p for 3 weeks. Treated mice were killed 4 weeks post-treatment. Ascites volume, tumor weight, CA125 levels from ascites and survival of animals were assessed. The expression of MUC1, CD31, Ki-67, TUNEL and apoptotic proteins in tumor xenografts was evaluated by immunohistochemistry. MAb C595 alone inhibited i.p. tumor growth and ascites production in a dose-dependent manner but did not obviously prevent tumor development. However, combination test significantly reduced ascites volume, tumor growth and metastases, CA125 levels in ascites and improved survival of treated mice compared with single agent-treated mice, combination control or vehicle control-treated mice (*P*<0.05). The data was in a good agreement with that from cultured cells *in vitro*. The mechanisms behind the observed effects could be through targeting MUC1 antigens, inhibition of tumor angiogenesis, and induction of apoptosis. Our results suggest that this combination approach can effectively reduce tumor burden and ascites, prolong survival of animals through induction of tumor apoptosis and necrosis, and may provide a potential therapy for advanced metastatic EOC.

## Introduction

Epithelial ovarian cancer (EOC) is the most lethal gynecological malignancy and the fifth most common cause of cancer-related deaths in women in the United States, resulting in an estimated 21,880 new cases and 13,850 deaths in 2009 [1]. Patients with advanced disease have a response rate of more than 80% following surgery and adjuvant chemotherapy with platinum-taxane, with a median progression-free interval of 18 months [2]. Unfortunately, the cancer will recur in the majority of these patients, and the overall 5-year survival rate for patients with advanced stage disease is only 23–30% [3]. Conventional cancer chemotherapy often results in severe side effects related to non-specific modes of action. Studies evaluating various cytotoxic agents in recurrent EOC have found response rates of 10–28% with an accompanying progressive increase in the number of drug-resistant tumors [4]. Thus, novel therapeutic strategies are urgently needed to improve the outcome for this deadly disease. A promising approach that may improve patient outcome is the use of monoclonal antibodies (MAbs) combined with traditional chemotherapy.

MUC1 is a large molecular weight transmembrane glycoprotein that is overexpressed in many carcinomas [5], [6] including EOC [7]–[9], and mediates signal transduction events that stimulate the motility, invasion, and metastasis of cancer cells [10]. MUC1 is over-expressed on 90% of EOC cell surfaces [7], [8]. The enhanced levels of MUC1 expression by cancer cells may mask extra-cellular domains from immune surveillance, conferring a survival advantage on malignant cells and playing an important role in the ability of tumors to invade and metastasize [11]. Thus, tumor-associated MUC1 is a promising molecular target for a novel therapy for EOC patients.

C595 is an IgG_3_, murine MAb raised against the protein core of human MUC1 (urinary epithelial mucin1) [12]. Epitope mapping has shown that C595 recognizes a tetrapeptide motif (RPAP) within the protein core of MUC1 mucin that contains a large domain of multiples of a highly conserved 20-amino-acid repeat sequence (PDTRPAPGSTAPPAHGVTSA) [12], [13]. MAb C595 has been labeled with γ-emitting radioisotope (^111^In) to test its capacity for cancer localization and identification in 19 patients with a clinical suspicion of ovarian malignancy, and achieved final accuracies of 79% and 64% compared to magnetic resonance imaging and ultrasound in relation to the final tumor histology [14]. After labeling with α-emitter (^213^Bi), ^213^Bi-C595 α-conjugate (AC) has been used to target single prostate [15], pancreatic [16] and ovarian cells [17] *in vitro* and regress pancreatic subcutaneous xenografts *in vivo* [18]. These results support the hypothesis that the MAb C595 is useful either alone or in combination with other therapies to improve the treatment of the advanced EOC.

Although paclitaxel is used in first-line therapy, its analogue docetaxel (DTX) has advantages, including slower cellular efflux and higher solubility, allowing for higher intracellular concentrations [19]. In clinical trials, DTX resulted in equivalent response rates and has shown activity against paclitaxel-refractory carcinomas [20], [21]. DTX has demonstrated significant activity in both pre-clinical and clinical studies for the treatment of numerous solid malignancies including EOC [22]–[25]. It is plausible that DTX may become part of the first-line therapy for EOC. DTX combined with a platinum compound (such as carboplatin) has become the systemic chemotherapy of choice for primary EOC, with high efficacy. However, dose-related toxicity and the eventual development of resistance are major issues requiring attention in a gynecologic oncology setting. Ultimately most of these patients will die of metastatic disease. Maintaining low drug levels in the systemic circulation through localized delivery can consequently decrease toxic side effects, and increase local drug concentrations in the peritoneum, where ovarian tumors and ascites reside. This can be achieved through i.p. administration. The National Cancer Institute has recommended that i.p. chemotherapy be considered for the treatment of advanced ovarian cancer [26]. Combination therapy specifically employing strategies such as a chemotherapeutic agent plus an antibody through i.p. administration may efficiently reduce dose-limiting toxicity and improve treatment efficacy.

In a recent study, we demonstrated that MAb C595 alone could kill EOC cells in a dose-dependent manner; this killing was also dependent upon MUC1 expression levels. Low-dose MAb C595 combined with DTX increased EOC sensitivity to the chemotherapy drug and reduced the dose required [27]. In this study, we hypothesized that this combination treatment can effectively work in an *in vivo* EOC animal model. We found that MAb C595 could inhibit i.p. tumor growth and ascites production in the OVCAR-3 mouse xenograft model and enhance the therapeutic efficacy of DTX in a concentration-dependent manner, and that after i.p. injection, this combination treatment (test) (MAb C595 and DTX) could markedly reduce tumor burden and ascites and consequently prolong the survival of treated animals. Our results suggest that this novel combination holds promise as a potential therapy for the treatment of advanced metastatic EOC.

## Materials and Methods

### Drug

DTX was purchased from Sigma-Aldrich, Pty Ltd, Castle Hills, NSW, Australia. The drug was first diluted in [hydroperoxymethyl cellulose (HPMC) prepared as 0.5% in PBS] and stored at 4°C for use.

### Antibodies

MAb C595 was kindly provided by Nottingham University (Nottingham, UK). Mouse anti-human IgG_3_ isotype control MAb was purchased from Zymed Laboratories Inc (South San Francisco, CA, USA). Rabbit anti-human Ki-67, caspase-3 (active), and PARP-1 (cleaved p85) MAbs were provided by Epitomics (Burlingame, CA, USA). Rat anti-mouse CD31 MAb was purchased from BD Pharmingen (Bedford, MA, USA). Swine anti-goat, -mouse, -rabbit IgG/biotinylated, rabbit anti-rat IgG/biotinylated, streptavidin/horseradish peroxidase (HRP) and mouse IgG_1_ negative control MAb were purchased from Dakopatts (Glostrup, Denmark).

### Cell line and animal model

For all experiments, 6∼8 weeks old female nude athymic BALB/c nu/nu mice (Animal Resources Centre, Perth, Western Australia) were used. The mice were housed and maintained in laminar flow cabinets under specific pathogen-free conditions in facilities approved by the University of New South Wales (UNSW) Animal Care and Ethics Committee (ACEC). This study was approved by ACEC, UNSW (ID: 08/110A). Animals were kept at least 1 week before experimental procedure.

The primary OVCAR-3 EOC cell line was obtained from the American Type Culture Collection (Manassas, VA, USA), and the sub-line of OVCAR-3 was selected and successfully established in an i.p. xenograft model using nude mice in our laboratory [28]. This selection can increase the tumorigenicity of the OVCAR-3 cells *in vivo*. Briefly, viable OVCAR-3 cells (5×10^6^/500 µL in DPBS) were injected i.p. using a 1 mL syringe with a 26-gauge needle and allowed to grow. Tumor progression was documented once weekly for 10 weeks by measurements of abdominal circumference for abdominal increase using a soft ruler. Upon sacrifice, tumors were removed for weighting and proceeding for histological testing.

### Treatment protocols

Toxicity studies in nude mice without tumors: Toxicity studies were performed to determine the maximum tolerance dose (MTD) in mice for single MAb C595, mouse IgG_3_ negative control MAb, DTX, combination test (MAb C595 and DTX) and combination control (negative MAb IgG_3_ and DTX). The dose-tolerance relationship was examined in nude mice (without tumors) for a single i.p. administration of MAb C595, DTX and combination treatment (test) compared with combination control treatment. Groups of five mice received a total injected concentration of 1, 5, 10, 15, 20 mg/kg of MAb C595, or 3, 5, 7, 10, 15 mg/kg of DTX as well as combined MAb C595 (1/3 of MTD i.e 5 mg/kg) or 5 mg/kg MAb IgG_3_ with 3, 5, 7, 10 mg/kg of DTX for 3 weeks. The selected doses of MAbs were based on the MTD of single of MAb C595 and DTX. Mouse weights were compared with those at day 0 (first day of treatment administration) to determine percentage weight change. The dose-limiting toxicity was defined as end points: 15% loss of body weight or distressed behavior (i.e., loss of appetite and activity, hunched posture). The MTD was defined as the highest dose at which one third of the cohort reached dose-limiting toxicity end points [29]. After 10 weeks, healthy mice were euthanized. The following experiments were based on the MTD from the toxicity studies.

Efficacy studies in OVCAR-3 EOC animal model: After the development of ascites, the peritoneal cavity was washed with 2 mL of sterile normal saline. The peritoneal contents were mixed by managing gently and then completely aspirated [28]. Mice were then randomly distributed into either the MAb C595 or DTX alone single treatment group or the combination test (MAb C595 and DTX) and combination control (MAb IgG_3_ and DTX) group (n = 10, per group). Different treatments were initiated immediately after aspiration of the ascites fluid.

a). For single MAb C595 treatment, low dose (LD) (5 mg/kg) or high dose (HD) (15 mg/kg) MAb suspended in 1 mL of saline, and the same volume of saline with 15 mg/kg of mouse MAb IgG_3_ (control) were administered once/week for 3 weeks.

b). For single DTX treatment, LD (5 mg/kg) or HD (10 mg/kg) DTX suspended in 1 mL of HPMC (prepared as 0.5% in PBS) as a vehicle and the same volume of HPMC as a control.

c). For the combination test (MAb C595 and DTX) and combination control (MAb IgG_3_ and DTX) groups, the following was used. Combination test included one single dose of MAb C595 (1/3 dose of MTD, i.e. 5 mg/kg suspended in 0.5 mL of saline) combined with one single dose of DTX (10 mg/kg DTX suspended in 0.5 mL of HPMC). Combination control included one single dose of MAb IgG_3_ (5 mg/kg suspended in 0.5 mL of saline) combined with one single dose of DTX (10 mg/kg DTX suspended in 0.5 mL of HPMC). The combination treatment (test and control) was administrated sequentially from MAb C595 or MAb IgG_3_ to DTX.

All treatments were performed i.p. and intended duration of treatment was 4 weeks based on our previous studies. After treatments, if an animal's abdominal circumference reached 9.5 cm or if they were expected to become moribund within a short time, animals were euthanized. Survival time of each animal was calculated as the number of days elapsed between initiation of treatment and euthanasia. At the end of the experiments, mice were euthanized by cervical dislocation while under urethrane anesthesia.

### Sample collection

Before euthanasia, a blood sample was collected through cardiac puncture under anesthesia. At the end of experiments, 2 mL of physiologic saline were injected i.p. and the peritoneal cavity was completely washed and aspirated for CA125 detection. Ascites volumes in the peritoneal cavity were recorded. The ascites fluid wash, tumors dissected from the peritoneal cavity, and the plasma, were all stored at −80°C for subsequent analysis. Cumulative actual volume of ascites collected after each aspiration was calculated by subtracting the 2 mL from the total volume collected.

### CA125 levels in ascites fluid

Tumor marker levels (CA125 Ku/L) in cell-free ascites fluid from single MAb C595, DTX and combination treatments (test and control) were determined by an ELISA assay following the instructions at the St George Hospital Biochemistry Laboratories. The concentration of CA125 in the ascites was normalized to the actual volume of ascites collected.

### Mouse tissues and histology

The tumor tissues from single MAb, DTX, combination or vehicle control-treated animals were either immediately snap frozen for frozen sections or fixed in 10% formalin for 24 hours, embedded in paraffin block for H&E staining and immunohistochemistry. Five-micrometer frozen sections of fresh tumor samples were used for CD31 immunostaining. For toxicity studies, relevant mouse organs such as kidney, liver, heart and bone marrow were collected, formalin-fixed and sent for pathologic examination (IDEXX Laboratories, Sydney, Australia).

### Hematological toxicity and renal function examination

To determine hematological toxicity, 200 µL of blood in each mouse was collected into K3 EDTA and Z serum gel minicollect tubes (Greiner Bio-one, Germany) via the saphenous vein and Microvette (SARSTEDT, Germany) before treatment and at 2 and 3 weeks post single or combination injection. Hematological analyses of white blood cell (WBC), lymphocytes, red blood cell (RBC), and platelet counts were performed. Blood was obtained at the end of experiments for biochemical analysis of serum for renal functions.

### Immunohistochemistry

Standard immunoperoxidase procedures were used to visualize MUC1, Ki-67, caspase-3 (active) and PARP-1 (cleaved p85). Briefly, paraffin sections were deparaffinised in xylene, followed by a graded series of alcohols (100%, 95%, and 75%) and re-hydrated in water followed by Tris-buffered.

Saline (TBS) (pH 7.5). Slides were subsequently immersed in boiling 0.01 M citrate buffer (pH 6.0) for 15 min to enhance antigen retrieval, treated with 3% hydrogen peroxide and then incubated with primary MAbs: MUC1 (1∶500 dilution), Ki-67 (1∶50 dilution), caspase-3 (active) (1∶100 dilution) and PARP-1 (cleaved p85) (1∶100 dilution), respectively overnight (o/n) at 4°C. After washing with TBS, slides were incubated with swine anti-goat, mouse, rabbit biotinylated IgG second antibody (1∶150 dilution) for 45 min at room temperature (RT), and then with avidin/ horseradish peroxidase (HRP) solution (1∶300 dilution) for 30 min at RT. Sections were finally developed with 3,3′ diaminobenzidine (DAB) substrate solution (Sigma-Aldrich, Pty Ltd, Castle Hills, NSW, Australia), then counterstained with hematoxylin; positive cells appeared brown. Control slides were treated in an identical manner, and stained with the isotype MAb or omission of the primary antibody as a negative control. Positive controls were chosen depending on different MAbs, colon carcinoma tissue for MUC1, tongue tissue for Ki-67 and DTX-treated OVCAR-3 cell line for caspase-3 (active) and PARP-1 (cleaved p85).

For CD31 staining, the frozen sections were thawed, air-dried and fixed in cold acetone for 10 min at RT. After a quick air dry and washing with TBS, the sections were incubated with rat anti-mouse CD31 MAb (1∶100 dilution) o/n at 4°C. After rinsing with TBS, sections were incubated with rabbit anti-rat biotinylated IgG (1∶200 dilution) for 45 min, and then with conjugated streptavidin/HRP (1∶200 dilution) for another 30 min. Sections were developed by using DAB solution (Sigma-Aldrich, Pty Ltd, Castle Hills, NSW, Australia), and counterstained with hematoxylin. Control slides were treated in an identical manner, and stained with the isotype MAb or omission of the primary antibody as a negative control.

### TUNEL assay for apoptotic cells *in vivo*

Apoptosis was assessed on tumor xenograft tissues using the TUNEL method with the TdT-fragEL in situ apoptotic detection kit (Calbiochem, San Diego, CA, USA) according to the manufacturer's instructions. The specificity of TUNEL reactivity was confirmed with appropriate negative (TdT omitted from the labeling mix) and positive (treated HL-60 slides provided by the company) controls. Slides were examined using a Leica light microscope (Nussloch, Germany).

### Assessment of immunostaining

Staining intensity (0–3) was assessed using light microscopy (Leica microscope, Germany) at a ×40 objective as − (negative), + (weak), ++ (moderate), and +++ (strong). Evaluation of tissue staining was done, independently, by two experienced observers (LW and HMC). All specimens were scored blind and an average of grades was taken. If discordant results were obtained, differences were resolved by joint review and consultation with a third observer, experienced in immunohistochemical pathology.

### Statistical analysis

All numerical data were expressed as the average of the values obtained, and the standard deviation (SD) was calculated. The data from treated and control groups were compared using the two-tail student's t test. All *P* values were 2-sided. One way ANOVA, followed by the Dunnett's post hoc test was performed to determine significant differences in mean mice weight changes in toxicological studies. Survival was calculated as the number of days lapsed between initiation of treatment and euthanasia, and % mice surviving was the number of animals remaining in each group (×10) at the end of each week following initiation of treatment. *P*<0.05 was considered significant. All statistical analyses were performed using the GraphPad Prism 4.00 package (GraphPad, San Diego, CA, USA).

## Results

### Toxicological evaluation of single MAb C595, DTX and combination treatments

**Figure 1:**
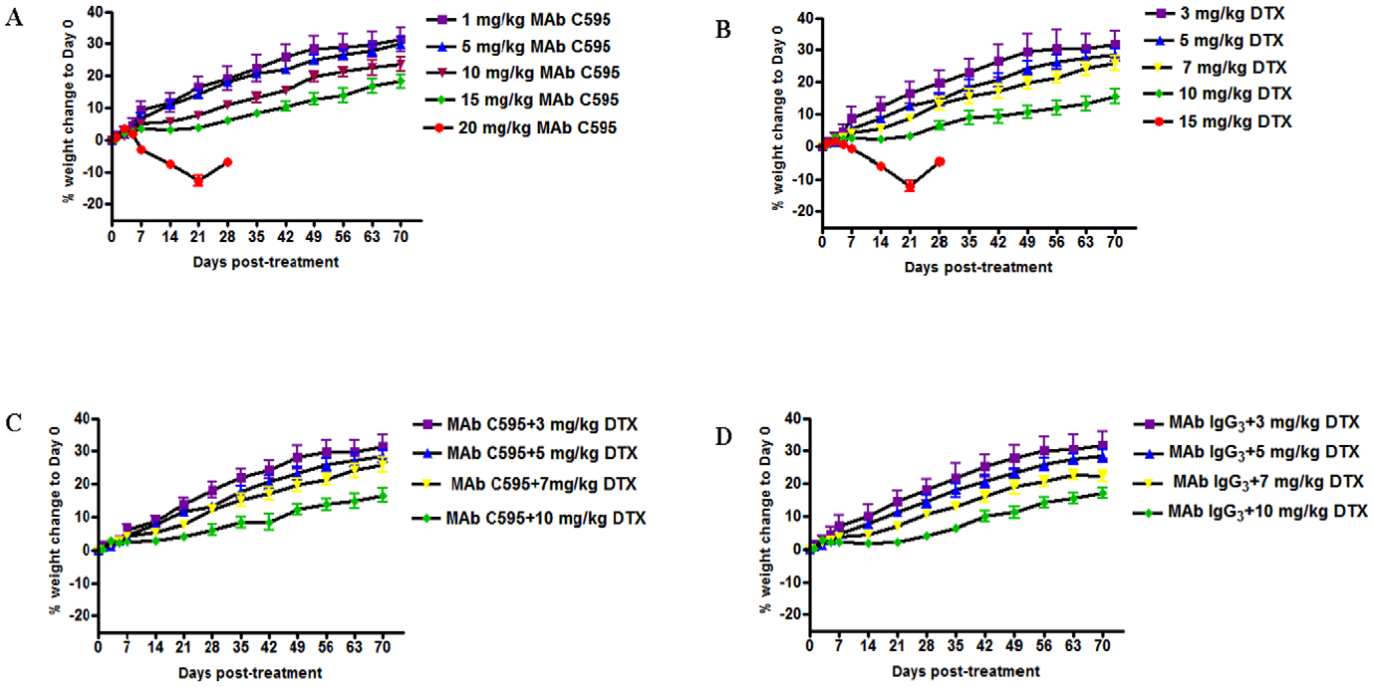
Dose-tolerance studies for escalating single-dose administration of single or combination treatments for the first 3 weeks in nude mice without tumors. Average percentage weight changes compared with day 0 (i.e., day of single or combination administration). A. Dose-tolerance relationship in mice by single MAb C595. ▪: MAb C595 (1 mg/kg); ▴: MAb C595 (5 mg/kg); ▾: MAb C595 (10 mg/kg); ♦: MAb C595 (15 mg/kg); •: MAb C595 (20 mg/kg). The significant difference was found between 20 mg/kg of MAb C595-treated group and other MAb C595-treated groups (5–15 mg/kg) (*P*<0.05). B. Dose-tolerance relationship in mice by single DTX. ▪: DTX (3 mg/kg); ▴: DTX (5 mg/kg); ▾: DTX (7 mg/kg); ♦: DTX (10 mg/kg); •: DTX (15 mg/kg). The significant difference was found between 15 mg/kg of DTX-treated group and other DTX-treated groups (5–15 mg/kg) (*P*<0.05). C. Dose-tolerance relationship in mice by combination test [MAb C595 (5 mg/kg)+DTX (3–10 mg/kg)]. ▪: MAb C595+DTX (3 mg/kg); ▴: MAb C595+DTX (5 mg/kg); ▾: MAb C595+DTX (7 mg/kg); ♦: MAb C595+DTX (10 mg/kg). D. Dose-tolerance relationship in mice by combination control [MAb IgG_3_ control (5 mg/kg)+DTX (3–10 mg/kg)]. ▪: MAb IgG_3_+DTX (3 mg/kg); ▴: MAb IgG_3_+DTX (5 mg/kg); ▾: MAb IgG_3_+DTX (7 mg/kg); ♦: MAb IgG_3_+DTX (10 mg/kg). Points, mean (n = 5 in each group); bar, SD.

Single-dose administration of MAb C595 at 1, 5, 10, 15 mg/kg and DTX at 3, 5, 7, 10 mg/kg for the first 3 weeks did not reach toxicity end points at 70 days post-treatment (**Fig. 1A and B**). A single administration of control MAb IgG_3_ at 15 and 20 mg/kg for the first 3 weeks did not reach toxicity end points at 70 days post-treatment (data not shown). Mice treated with 20 mg/kg MAb C595/week or 15 mg/kg DTX/week started to lose weight and were euthanised 4 weeks post treatment because of signs of distress; histopathological examination indicated mild nephropathy in these groups. The results suggest that the MTD for single dose MAb C595 lies between 15 and 20 mg/kg while the MTD for a single administration of DTX lies between 10 and 15 mg/kg.

For the combination treatments, we used 1/3 MTD MAb C595 (i.e. 5 mg/kg) as a test and 5 mg/kg of negative MAb IgG3 as a control combined with a range of DTX (1∼10 mg/kg) to further evaluate the toxicity of the combination treatments. No toxicity was found in the combination treatments (Fig. 1C and D). There were no macroscopic signs of chronic toxicity to major organs for any mice. Leukocyte counts were depressed in peripheral blood in treated mice at 2 weeks post injection, with recovery occurring by 4 weeks and normal hematology was seen at 70 days. These results suggest that combination treatments with MAb C595 and DTX or with control MAb IgG_3_ and DTX could be safely used in animal models to study efficacy.

### Effect of single MAb C595 and DTX on tumor growth inhibition and ascites production

**Figure 2:**
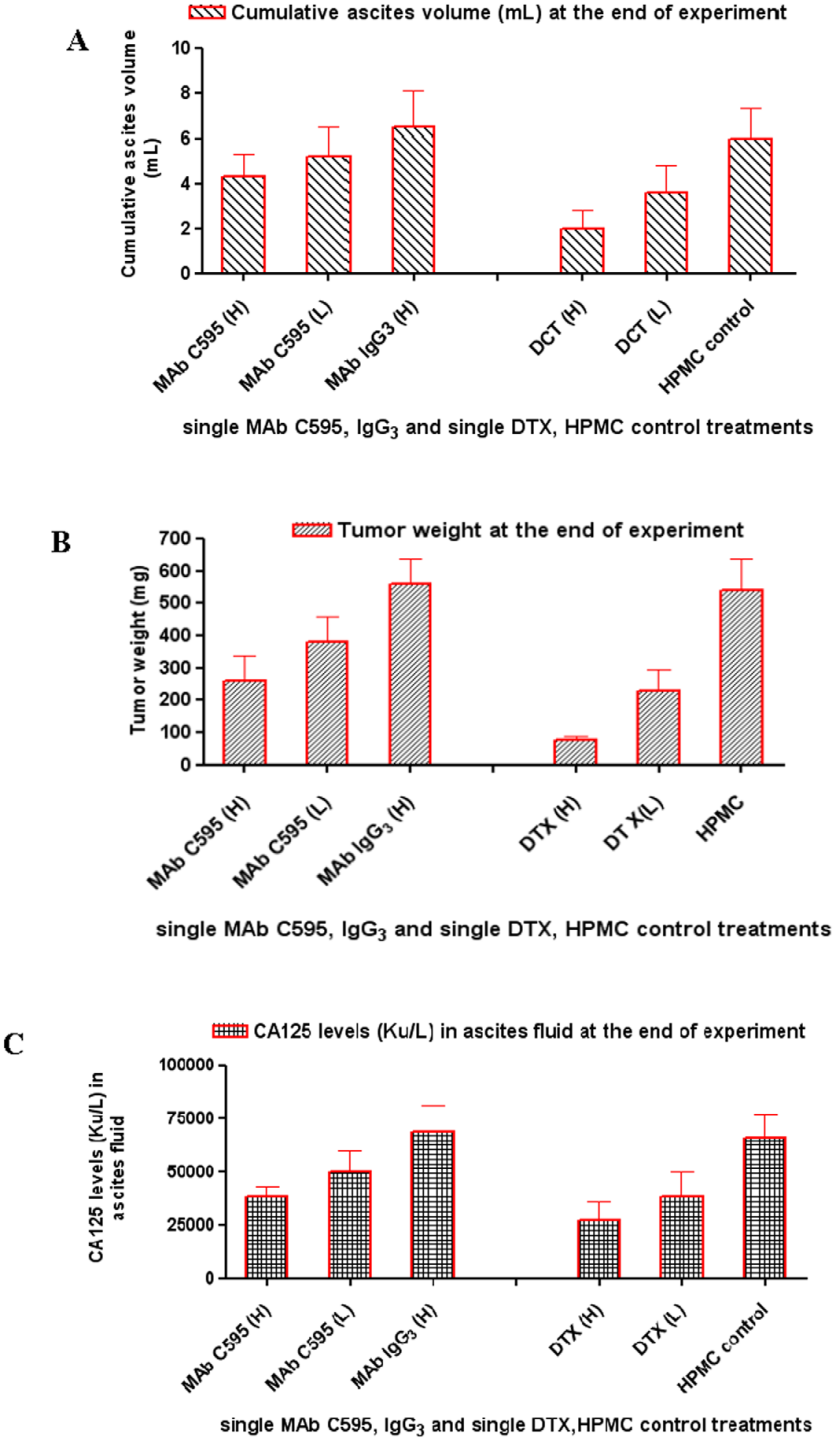
Cumulative ascites volume, tumor weight and CA125 levels at the end of experiments after single MAb C595 and DTX treatment in OVCAR-3 animal model. Following euthanasia, the peritoneal cavity of each mouse was washed with 2 mL of normal saline, and after aspiration, the volume of ascites present was recorded. A. Cumulative volumes of ascites collected from each animal from initiation of therapy [MAb C595 (H and L), MAb IgG_3_, DTX (H and L) are shown. The obvious difference was seen between DTX(H)-treated group and others-treated group (*P*<0.05). B. Tumor weights (mg) at the end of experiments after single MAb C595, MAb IgG_3_, DTX or vehicle treatments with different doses. The tumor weigh was obviously lower in MAb-treated and DTX-treated groups compared to MAbIgG_3_-treated and HPMC-treated control groups (*P*<0.05). C. Effect of single MAb C595, MAb IgG_3_, DTX or vehicle on suppressing the increase in the tumor marker CA125 (CA125 Ku/L) in the ascites fluid (peritoneal wash) at the end of experiments. The level of CA125 was obviously lower in MAb C595 (H)-treated and DTX-treated groups compared to MAbIgG_3_-treated and HPMC-treated control groups (*P*<0.05). H: high dose; L: low dose. Representative graphs are shown.

We firstly compared the antitumor activity of single MAb C595, MAb IgG_3_ control, single DTX and vehicle control (HPMC) following development of ascites and initial aspiration for 3 weeks. Cumulative volume of ascites fluid produced per animal is presented in Fig. 2A. Vehicle-treated mice continued to produce ascites at a more frequent rate and had to be aspirated repeatedly during the course of the treatment (28 days) while MAb C595-treated mice produced less ascites related to the dose of MAb C595 (4±1 mL for HD and 5±1 mL for LD, respectively) compared with MAb IgG3 control mice (7±2 mL) (Fig. 2A). DTX-treated mice developed ascites slowly compared with HPMC control (6±1 mL) and the production of ascites was related with the dose of DTX (2±1 mL for HD and 4±1 mL for LD, respectively) (Fig. 2A). The HD of DTX (10 mg/kg) significantly reduced the development of ascites (P<0.05, Fig. 2A). In addition to ascites production, we also evaluated the total tumor weight change in each group at the end of the experiments (4 weeks after treatment). The tumor weight change is consistent with the change of ascites production (Fig. 2B). Single MAb C595 only partially inhibited the growth of OVCAR-3 tumors, as evidenced by tumor weight of 380±80 mg in LD (5 mg/kg) and 260±76 mg in HD (150 mg/kg) versus 560±77 mg in mice receiving negative MAb IgG3 control (15 mg/kg) (P<0.05), respectively while single DTX obviously inhibited the growth of OVCAR-3 tumors, as evidenced by tumor weight of 230±66 mg in LD (5 mg/kg) and 75±14 mg in HD (10 mg/kg) versus 540±96 mg in mice receiving the same volume of HPMC (P<0.05), respectively (Fig. 2B). These results suggest that both MAb C595 and DTX can induce regression of OVCAR-3 tumor growth in a dose-dependent manner and that anti-tumor activity of DTX is more effective than that of MAb C595.

### Effect of combination of MAb C595 and DTX on tumor growth, ascites production and survival

**Figure 3:**
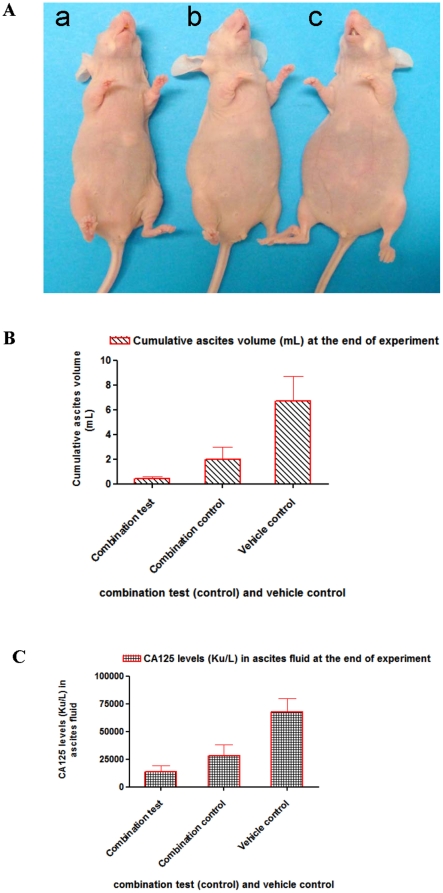
Cumulative ascites volume and CA125 levels at the end of experiments after combination test and combination control treatments. A. At the end of experiments, no obvious signs of ascites formation are seen in combination test (5 mg/kg MAb C595+10 mg/kg DTX)-treated mice (a); signs of ascites formation was found in combination control (5 mg/kg MAb C595+10 mg/kg DTX)-treated mice (b); obvious signs of ascites formation was found in vehicle (1/2 saline+1/2 HPMC)-treated mice (c). B. The cumulative ascites volumes from combination test, combination control or vehicle-treated mice are shown (*P*<0.05). C. Effect of combination test, combination control or vehicle on suppressing the increase in tumor marker level (CA 125 Ku/L) in the ascites fluid (peritoneal wash) at the end of experiments (*P*<0.05). Representative image and graphs are shown.

**Figure 4:**
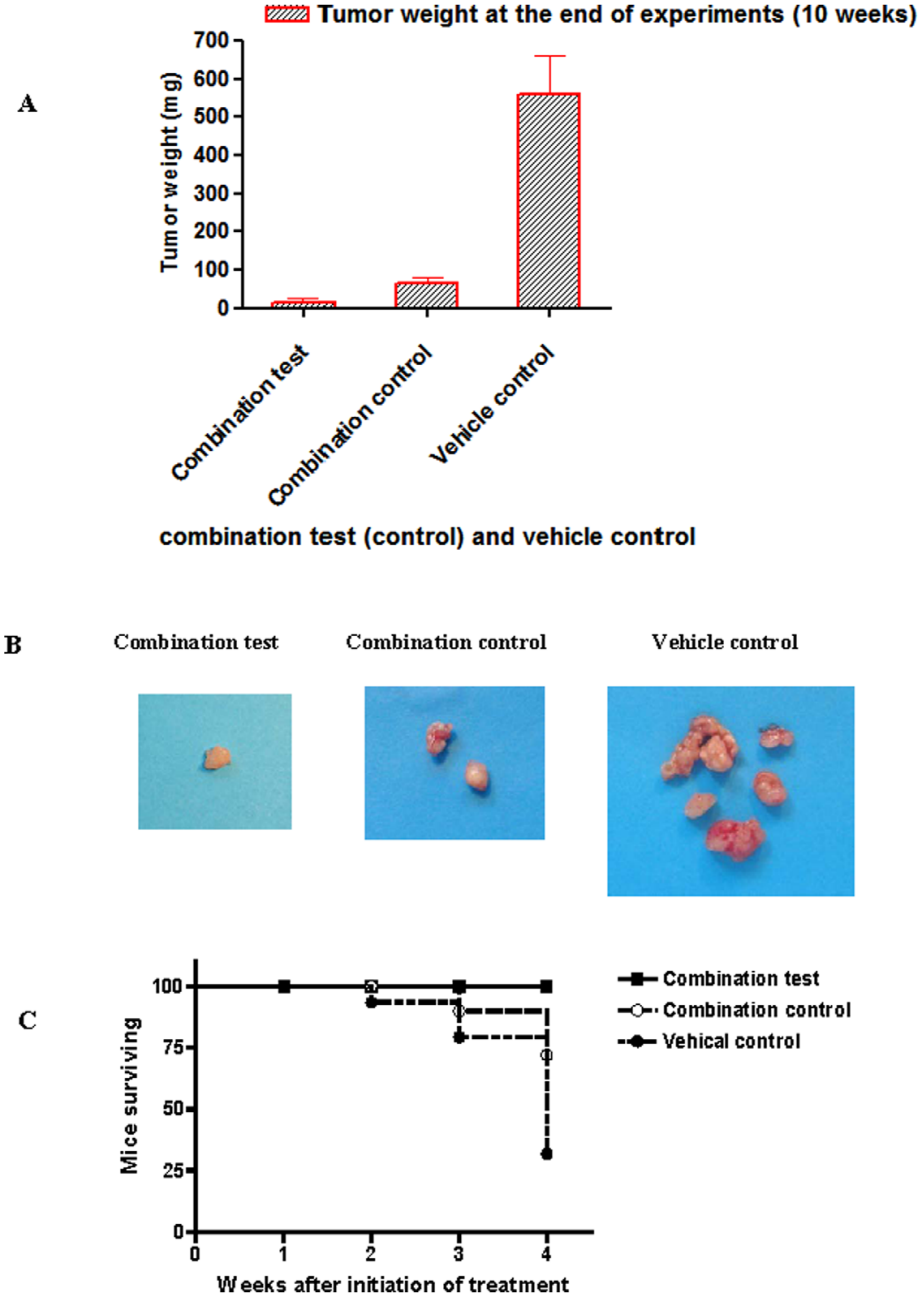
Tumor weight and survival curve in combination test (control). A. Tumor weight changes at the end of experiments after combination test, combination control or vehicle treatments with different doses (*P*<0.05). B. The tumor volume in combination test-treated mice was significantly lower than in vehicle control-treated mice (*P*<0.01). C. For all animals, the intended duration of treatment was 4 weeks. Mice (10 per group) were euthanized if due to ill health, they were expected to become moribund within a short time. Survival was calculated as the number of days lapsed between initiation of treatment and euthanasia, and % mice surviving was the number of animals remaining in each group (×10) at the end of each week following initiation of treatment. The survival rate of animals in the combination test group was much better than that in the combination control group or vehicle group (*P*<0.05). Representative images and graph are shown.

We then compared the antitumor activity of the combination of 1/3 MTD MAb C595 (5 mg/kg) or control MAb IgG_3_ (5 mg/kg) with one dose DTX (10 mg/kg) following development of ascites and initial aspiration for 3 weeks. The combination test (5 mg/kg MAb C595 and 10 mg/kg DTX) could significantly inhibit the development of ascites compared with combination control (5 mg/kg MAb IgG3 and 10 mg/kg DTX) and vehicle control (Fig. 3A). The pattern of ascites production in the combination control group is similar to that seen for a single 10 mg/kg DTX treatment as described above. Cumulative volume of ascites fluid produced per animal are presented for combination test (0.4±0.2 mL), combination control (2±1 mL) and vehicle control (7±2 mL) in Fig. 3B (P<0.05). Four weeks after treatment, combination test treatment significantly inhibited the growth of OVCAR-3 tumors, as evidenced by tumor weight of 15±11 mg versus 65±16 mg in the combination control group and 560±100 mg in the vehicle control group (P<0.05), respectively (Fig. 4A and B). The survival of animals was much better in the combination test group than the combination control and vehicle control groups (P<0.05) (Fig. 4C).

### Single or combination treatment affects tumor marker CA125 levels

At the end of experiments, change in mean CA125 levels for single MAb C595 in LD was not significantly different compared to MAb IgG_3_ control, whereas the change in mean CA125 levels for single CA125 in HD or single DTX was obviously different compared to MAb IgG_3_ or HPMC control, respectively (*P*<0.05) (**Fig. 2C**). It is evident that the combination test group had significantly inhibited CA125 levels compared with the combination control group (*P*<0.05) or vehicle control group (*P*<0.01) (**Fig. 3C**). At euthanasia, the CA125 vehicle values were 68,000±12,000 Ku/L, compared to the combination test and combination control of 14,030±5,022 Ku/L and 28,306±11,403 Ku/L, respectively. The reduction in cell-free ascites fluid CA125 levels is consistent with the reduction in ascites volume and tumor weight.

### Histological alterations in tumor xenografts after combination treatment

**Figure 5:**
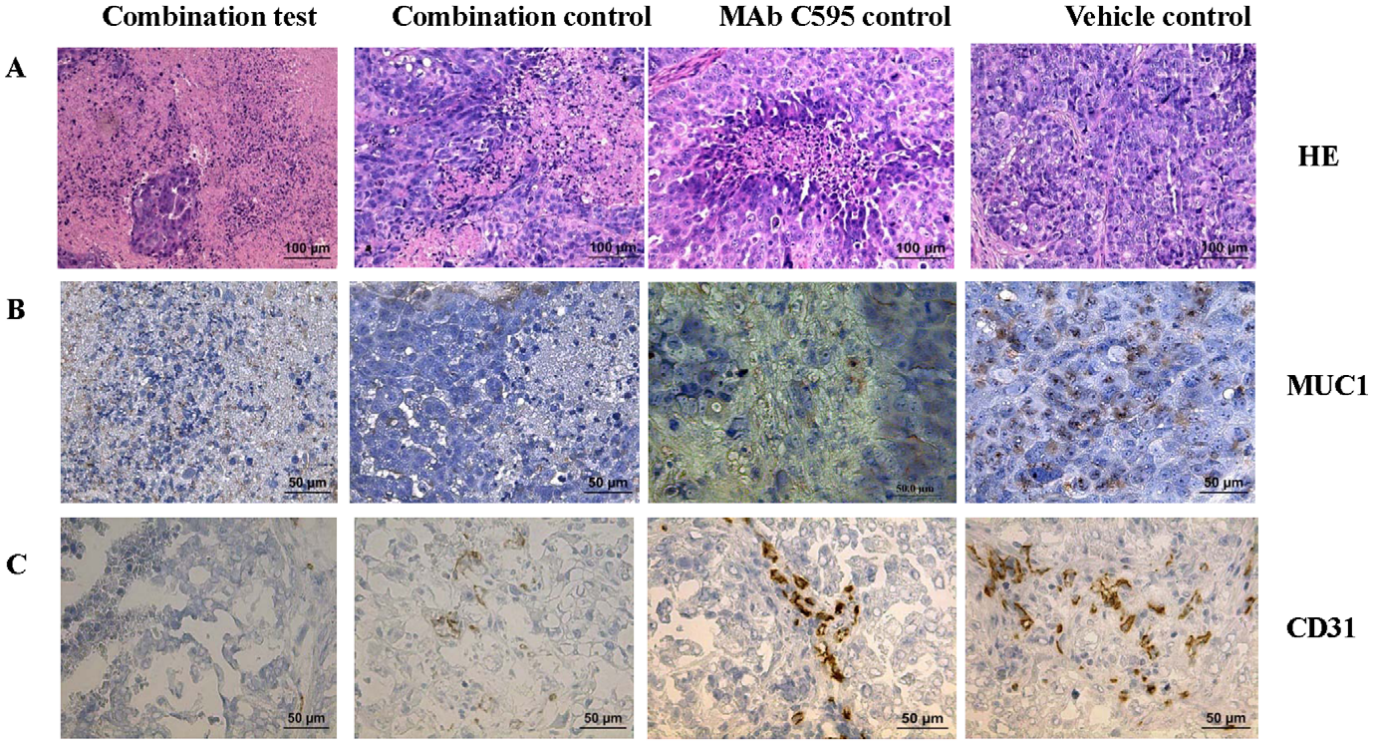
Representative images of histological changes, MUC1 and CD31 expression at the end of experiments after combination and control treatments. A. Obvious targeted lesions are shown in combination test; medium targeted are shown in combination control and MAb C595 control while non targeted lesions are shown in vehicle control. B. Very low MUC1 expression is shown in combination test; low MUC1 expression is shown in combination control and MAb C595 control while high MUC1 expression is shown in vehicle control (*P*<0.05). C. Markedly reduced CD31 expression is shown in combination test; very low CD31 expression is shown in combination control; very low to medium CD31 expression is shown in MAb C595 control while high CD31 expression is shown in vehicle control (*P*<0.05). Brown color staining indicates positive while blue hematoxylin stains nuclei. Magnification ×20 in A; Magnification ×40 in B, C. Representative images are shown.

To compare the histology of each group, we harvested tumors from combination-treated, MAb C595 control-treated and vehicle control-treated mice at the end of the experiments and stained paraffin sections with H&E. As assessed by light microscopy (Fig. 5), many targeted lesions were found to be considerably less cellular and composed primarily of acellular material in combination test-treated xenografts and cells were most commonly found in small islands whereas less targeted lesions were found in combination control-treated xenografts (Fig. 5A). In contrast, tumors from mice given vehicle control consisted of tightly packed cells, with many blood vessels apparent within the tumors (Fig. 5A). Low number of targeted lesions was found in single MAb C595-treated mice (Fig. 5A). These results indicated a very large difference in tumor cell burden among different treatments and showed that combination test treatment obviously affected tumor growth.

### MUC1 changes after combination treatment

At the end of experiments, the percentage of MUC1 positive staining cells visualized using MAb C595, in tumor xenografts from combination test, combination control, MAb C595 control and vehicle control groups was <5%, 10∼16%, 30–40% and 70∼80%, respectively (**Table 1**). The expression of MUC1 in combination test was remarkably reduced compared with that in combination control, MAb C595 control and vehicle control (*P*<0.05) (Fig. 5B). These results indicate that combination test including MAb C595 can specific target cancer surface MUC1 and reduce MUC1 expression.

**Table 1:** The intensity of immunohistochemical staining of MUC1, CD31, Ki-67, TUNEL, Caspase-3 (Active) and PARP-1 (Cleaved p85) in tumor xenografts from combination test, combination control, MAb C595 control and vehicle control.

Tumor (n = 5)	MUC1	CD31	Ki-67	TUNEL	Caspase-3 (A)	PARP-1 (C)
Combination test*	+	+	+	+++	++∼+++	++∼+++
	<%5	3–5/area	5–10%	7–10/area	74–88%	80–90%
Combination control	**+**	**+**	**+**	**++**	**+∼++**	**+**
	10–16%	8–12/area	15–20%	4–6/area	35–48%	30–45%
MAb C595 control	**+∼++**	**++∼+++**	**+∼++**	**++∼+++**	**+**	**+**
	30–40%	15–20/area	25–36%	3–5/area	15–23%	10–16%
Vehicle control	**++∼+++**	**++∼+++**	**++∼+++**	**+**	−	−
	70–80%	30–40/area	72–85%	0/area	0	0

All sections were prepared at the end of experiments.

Abbreviations: −, negative; +, weak; ++, moderate; +++, strong.

*indicates that obvious difference was found between combination test and controls.

**A**: active; **C**: Cleaved p85.

### Assessment of tumor vascular density after combination treatment

Representative micrographs from sections of excised tumor xenografts of combination test, combination control and vehicle control-treated mice immunostained with MAb CD31 are shown in Fig. 5C. The numbers of positive staining cells/area in combination test, combination control, MAb C595 control and vehicle control groups were 3∼5, 8∼12, 15∼20 and 30∼40%, respectively. The staining results are summarized in Table 1. A marked reduction in the number of CD31^+^ blood vessels was seen in tumors from combination test group compared to combination control, MAb C595 control and vehicle treated groups (*P*<0.05) (Fig. 5C). These results suggest that combination test targets angiogenesis in OVCAR-3 model.

### Assessment of cell death, proliferation and apoptotic proteins after combination treatment

**Figure 6:**
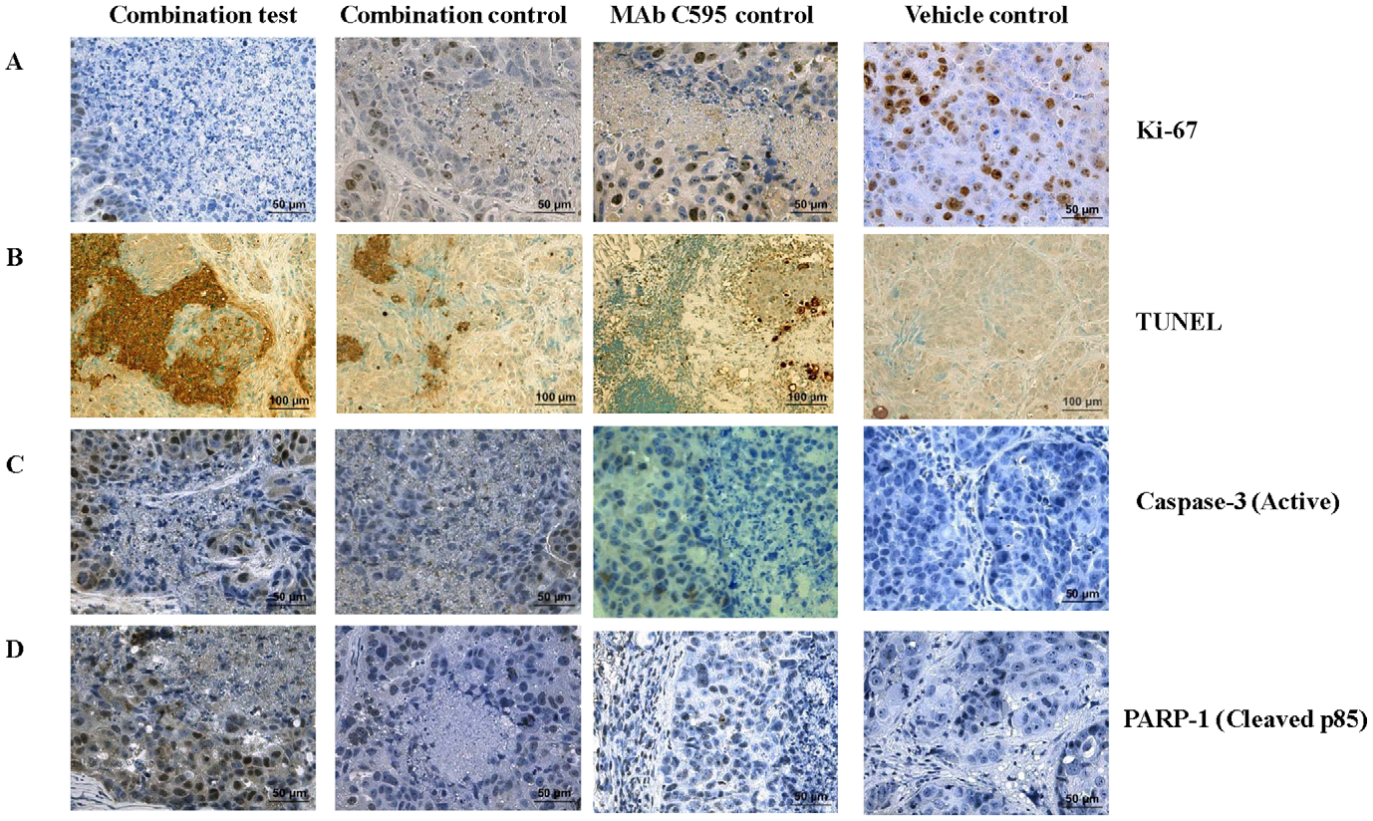
Representative images of Ki-67, TUNEL, Caspase-3 (Active) and PARP-1 (Cleaved 85) at the end of experiments after combination and control treatments. A. Very low Ki-67 expression is seen in the combination test; reduced Ki-67 expression in the combination control and MAb C595 control while high Ki-67 expression is seen in the vehicle control (*P*<0.05). B. Obvious TUNEL-positive cells are shown in combination test; some TUNEL-positive cells are shown in the combination control and MAb C595 control while no TUNEL-positive cells are seen in the vehicle control (*P*<0.05). C. High caspase-3 (active) expression is shown in combination test; low caspase-3 (active) expression is shown in combination control and MAb C595 control while negative caspase-3 (active) expression is shown in vehicle control (*P*<0.05). D. High PARP-1 (cleaved p85) expression is shown in combination test; low PARP-1 (cleaved p85) expression is shown in combination control and MAb C595 control while negative PARP-1 (cleaved p85) expression is shown in vehicle control (*P*<0.05). The brown color indicates nuclear staining in Ki-67, caspase-3 (active) and PARP-1 (cleaved p85), while blue indicates nuclear staining with hematoxylin. In the TUNEL assay, the brown color indicates nuclear chromatin condensation and fragmentation with methylgreen nuclear staining. Magnification ×20 in B; Magnification ×40 in A, C, D. Representative images are shown.

To investigate the effect of combination treatment on tumor cell proliferation, tumor sections from nude mice were assessed for Ki-67 expression, which is an indicator of underlying cell DNA synthesis. Antibody against Ki-67 recognizes G1, S, G2 and M phases, but not the G0 cells out of the cell cycle [30]. After 4 weeks of treatment, very low numbers of Ki-67+ tumor cells were seen in combination test (5∼10%) mice, modest Ki-67+ tumor cells in combination control (15∼20%) and MAb C595 treated (25∼36%) mice, and many Ki-67+ tumor cells were seen in vehicle control-treated (72∼85%) mice (P<0.05), respectively (Table 1). Representative images are shown in Fig. 6A.

To investigate if the mechanisms involved in the induction of apoptosis in targeted lesions of tumor xenografts represented a phenotypic response of OVCAR-3 tumors, the TUNEL assay was performed. Representative results are shown in Fig. 6B. After treatment, the tumor cells in combination test-treated (7∼10/area) mice showed typical apoptotic cell morphology with nuclear chromatin condensation and fragmentation, and tumor cells in combination control-treated (4∼6/area) and MAb C595 control-treated (3∼5/area) mice showed fewer apoptotic cells whilst tumors treated with vehicle control-treated did not (P<0.05) (Fig. 6B, Table 1). To further confirm apoptosis as a main mode of cell death following treatment, we observed high levels of caspase-3 (active) and PARP-1 (cleaved p85) positive cells in combination test-treated (74∼90%) xenografts, low levels of caspase-3 (active) and PARP-1 (cleaved p85) positive cells in combination control and MAb C595-treated (30∼48%) xenografts and caspase-3 (active) and PARP-1 (cleaved p85) negative cells in vehicle control-treated xenografts (P<0.05) (Fig. 6C and D). The decline in ascites production, tumor weight and vascular density was associated with a rapid decrease in Ki-67 expression (cell proliferation) and increases in TUNEL-positive lesions and expression of apoptotic proteins: caspase-3 (active) and PARP-1 (cleaved p85). The staining results for Ki-67, TUNEL, caspase-3 (active) and PARP-1 (cleaved p85) are summarized in Table 1.

## Discussion

Our recent results demonstrated that MAb C595 alone could kill EOC cells in a dose-dependent manner and that low-dose MAb C595 combined with DTX increased the sensitivity of several EOC cell lines and induced apoptosis *in vitro* [27]. In the present study, we further investigated whether MAb C595, combined with DTX, would be more efficient than DTX alone in killing EOC tumors in a xenograft animal mode previously developed by our laboratory [28]. EOCs account for 90% of all ovarian cancers, and can spread directly to adjacent organs; ‘seeding’ of the peritoneal cavity is frequently associated with ascites formation, the most common feature of ovarian carcinoma, particularly serous carcinoma [31]. Intra-abdominal dissemination is the primary cause of death for patients with EOC, although the exact mechanisms involved in EOC progression remain unclear. The OVCAR-3 i.p. xenograft model we developed has 100% tumor take, and can produce ascites and mimic the EOC spread in the peritoneal cavity, and thus is an appropriate model to investigate novel anti-ovarian cancer therapies.

Budiu *et al* (2011) found that increased serum MUC1 and high anti-MUC1 antibody levels are potential prognostic biomarkers for poor clinical response and reduced overall survival in platinum-resistant or platinum-refractory ovarian cancer [32]. We recently demonstrated that MAb C595 is strongly positive in over 90% of late stage of EOC sections as well as human EOC cell lines from primary tumors and metastatic lesions, while no staining was found in normal ovaries [8], [27]. In the present study, we also demonstrated MUC1 expression on OVCAR-3 tumor xenografts using MAb C595 and confirmed the target antigens (MUC1) exist after OVCAR-3 cells seed in peritoneal cavity (see Fig. 5B). MUC1 is an important marker of malignancy and is a target for several immunotherapies currently under investigation [33]. Gulley *et al* (2008) reported that in a pilot clinical study vaccination with recombinant CEA-MUC-1-TRICOM poxviral-based vaccines were effective in the treatment of EOC [34]. These data suggest that MUC1 is an interesting therapeutic target for EOC therapy and that MAb C595 has the potential to target MUC1-positive cancer cells in an animal model and can increase sensitivity to current chemotherapeutic agents.

Based on our cytotoxicity study, we evaluated the anti-tumor effects of single MAb C595 in LD (5 mg/kg) and HD (10 mg/kg) after 3 weeks cell inoculation in OVCAR-3 model and found that both LD and HD only partially prevented the production of ascites and reduced tumor weight at the end of experiments compared with the same amounts of MAb IgG_3_ control. These results indicate that MAb C595 alone cannot effectively target OVCAR-3 tumors *in vivo* (see Fig. 2). From previous observations, we know that OVCAR-3 cells in an animal model can form solid tumors after 1 week inoculation and that the solid tumors attain large volumes after 3 weeks inoculation. Due to penetration problems, MAb C595 cannot effectively target solid tumors, although MAb C595 can effectively kill monolayer EOC cells *in vitro* [27].

The mechanisms of action of MAb C595 on MUC1-positive EOC cells in the current study are unclear. We do not know whether MAb C595 can be internalized in cancer cells and trafficked after binding the cell surface MUC1, similar to MAb J591 [35] or how it can inhibit MUC1 signalling via different signalling pathways. However, many factors, including antigen affinity and antigen density play important roles in the killing of targeted antigen-positive cells. One possible explanation may be that MAb C595 binding MUC1 either blocks or stimulates a particular cell membrane molecule (e.g. growth factor receptor) through its cytoplasmic tail, inhibiting tumor growth. Another possibility is that in the presence of MAb C595, MUC1 and EGFR can be alternatively trafficked, and enter the lysosomal degradation pathway, with subsequent enhanced degradation. This would imply that targeting MUC1 signalling pathways by MAb C595 may reduce cancer proliferation, migration and invasion of metastatic EOC cells. MAb C595 may also have a direct cytotoxic effect on cancer cells [27]. The efficacy of anti-MUC1 MAb C595 has been reported in oral squamous cell carcinomas (OSCCs), where MAb C595 induced complement-dependent cytotoxicity (CDC) and antibody-dependent cellular cytotoxicity (ADCC) to OSCC cells; this effect was strongly correlated with MUC1 expression [36]. Furthermore, a humanized anti-MUC1 MAb (huHMFG-1) induced strong ADCC to breast cancer cells and is currently being used in a clinical trial for breast cancer [37]. These results suggest that in the clinical context MAb C595 may also induce specific immune response to attach MUC1-positive EOC cells. In contrast, Thie *et al* (2011) recently demonstrated that a human anti-MUC1 scFv antibody reacted with tumor cells in more than 80% of 228 tissue sections of mamma carcinoma samples but showed no significant decrease in tumor growth or increase in the survival rates in mouse xenograft models using MCF-7 and OVCAR-3 tumor cells [38]. One of the reasons for the failure of the xenograft experiments is a low ADCC, as detected *in vitro* for MCF-7 cells [38]. Another possible reason may be that MAb C595 has a different epitope for binding to stimulate immune response compared to this human anti-MUC1 scFv antibody.

DTX is now considered the preferred chemotherapeutic agent for EOC [23], [39]. The most widely described mechanism by which DTX achieves this effect is through its activity as a mitotic spindle poison, disrupting microtubule dynamics and inducing G2/M cell cycle arrest, a downstream effect thought to be related to the phosphorylation of Bcl-2 [40]. As we expected, DTX could prevent the production of ascites and inhibit tumor growth in a dose dependent manner compared with vehicle control and single MAb C595 treatment (*P*<0.05, see Fig. 2). Because of MTD limitation and side-effects of DTX in clinical condition, we further investigated the effect of combination test with low dose (1/3 MTD, 5 mg/kg) MAb C595 and high dose DTX (MTD, 10 mg/kg) on OVCAR-3 tumor development and survival of animals. Our results indicate that this combination test can significantly prevent production of ascites, regress tumor growth and improve the survival of animals compared with the combination control (5 mg/ kg IgG_3_ and 10 mg/kg DTX) and vehicle control (*P*< 0.05, see Fig. 3). This finding suggests that the combination of MAb C595 and DTX is a promising therapy for the late stage, drug resistant and recurrent EOC disease.

CA125 is a biomarker which can be used to monitor EOC progression. It is a surface mucin-like glycoprotein antigen that is expressed in more than 95% of all nonmucinous stage III/IV EOCs [41]. The serum level of CA125 is well established as a highly useful surrogate for monitoring the response to treatment and confirming relapse in EOC [42], [43]. The CA125 from ascites in our study was produced from the secretion of OVCAR-3 xenograft tumors and can be used as a surrogate for monitoring EOC progression after single or combination treatment. This biomarker was used in the same model in our previous study, where its efficiency for monitoring EOC progression was demonstrated [28]. In the present study, the CA125 levels were consistent with the production of ascites, tumor growth and survival of animals with single and combination treatments, suggesting that the CA125 is a useful marker to monitor the response to the treatment or relapse in the OVCAR-3 animal model.

The response of tumor growth to the combination test treatment in this study was specific, and highly dependent on antigenic expression. At the end of the experiments, most of the tumor antigen expression (MUC1) in the lesions had disappeared after treatment with the combination test and only a few scattered debris were found positive in these areas. These results suggest that combination treatment can specifically target tumor-associated antigen (MUC1) positive cancer cells.

Angiogenesis promotes the recovery of tumors following cytotoxic attacks, and it provides adequate nutritional and oxygen supply essential for tumor proliferation and metastasis [44]. In fact, the angiogenic potential of a tumor is directly correlated with poor prognosis [45]. De Souza *et al* (2010) have recently demonstrated that continuous DTX abrogates EOC tumor cell proliferation and angiogenesis to the tumor microenvironment in a SKOV-3 xenograft model, leading to greater tumor cell death than intermittent DTX therapy [25]. The effect of MAb C595 on tumor angiogenesis is still unclear. In the present study, we found that microvessel density (MVD) (CD31 expression) in combination test-targeted lesions was much less than with control treatments. These results suggest that MAb C595 may have a similar anti-angiogenesis function as seen for DTX, and both MAb C595 and DTX may have a concordant effect on tumor angiogenesis. MAb C595 may increase the efficacy of DTX in anti-angiogenesis in this animal model. The exact mechanisms of the combination treatment on angiogenesis and vascular regression will be investigated in a future study.

In this study, we found scattered targeted lesions with destruction of tissue structure and cell debris (necrosis) after 4 weeks treatment with combination therapy; fewer lesions were seen in combination control and MAb C595 control groups at the same time point. Other non-targeted areas showed “normal” tumor structure. The tumors treated with vehicle control also showed features of untargeted tissue structure. These results suggest that combination test can specifically and effectively kill EOC cells in xenografts.

As described above, treatment with combination test could effectively prevent production of ascites, delay tumor growth, reduce the volume of tumor xenografts and CA125 levels in ascite fluids and reduce vascularity of the tumor tissues. While the potential mechanisms for this remain unclear, we conducted preliminary studies to determine whether apoptosis was involved. We recently demonstrated that the combined MAb C595 and DTX could induce high levels of TUNEL-positive cells in single monolayer cultured EOC cells [27], suggesting apoptosis as a possible mode of cell death. In the present study, the therapeutic efficacy of the combination therapy included apoptosis and necrosis, and the number of apoptotic cells (TUNEL-positive) and the caspase-3 (active) and PARP-1 (cleaved p85) positive cells in OVCAR-3 tumors increased while the number of proliferating cells (using the Ki-67 marker) decreased. Caspase-3 is a member of the apoptosis execution functional group of caspases, and is either partially or totally responsible for the proteolytic cleavage of many key proteins during apoptosis [46]. It is activated by proteolytic cleavage into two active subunits only when cells undergo apoptosis [47]. PARP-1 is a marker for apoptosis that can be immediately activated by DNA strand breaks; the degree of PARP-1 activation could be an important factor in the cell's ability to repair the damage and survive or to die [48]. PARP-1 activation following limited DNA injury could constitute a signal to activate the repair and cell cycle control machineries. Apoptosis contributes to tumor volume reduction and can aid drug penetration into tumors by decreasing tumor cell density and expanding the tumor interstitium [49]. These results suggest that the mechanisms of action of the combination test in OVCAR-3 animal model involve in apoptosis and related with caspase-3 and PARP-1apoptotic proteins.

In summary, we have demonstrated for the first time that combining MAb C595 and DTX can effectively inhibit intraperitoneal tumor growth and ascites production, and prolong survival of animals in a mouse xenograft EOC model. Furthermore, this combination treatment can target tumor-associated antigens (MUC1), reduce angiogenesis and induce apoptosis. This combination treatment may provide a basis for reducing the dose of DTX required, without adversely impacting on treatment efficacy. Consequently, this combination treatment may be a potent therapeutic agent against advanced, recurrent, metastatic EOC disease.
